# Southern Tick–Associated Rash Illness in Pediatric Patient, New York, USA, 2025

**DOI:** 10.3201/eid3209.260871

**Published:** 2026-09

**Authors:** Andrew S. Handel, Sophia Ahmed, Ilia Rochlin, Hwan Keun Kim

**Affiliations:** Stony Brook University Renaissance School of Medicine, Stony Brook, New York, USA (A.S. Handel); Stony Brook University, Stony Brook (S. Ahmed, I. Rochlin, H.K. Kim)

**Keywords:** ticks, STARI, *Amblyomma americanum*, lone star tick, erythema migrans, *Borrelia lonestari*, bacteria, tick-borne disease, vector-borne infections, zoonoses, New York, United States

## Abstract

Southern tick–associated rash illness (STARI) is a tickborne disease of unknown etiology characterized by an erythema migrans–like skin lesion. We report a STARI case in New York in a child after an *Amblyomma americanum* tick bite; we detected *Borrelia lonestari*, a disputed cause of STARI, in the tick.

Southern tick–associated rash illness (STARI) is a tickborne disease of unknown etiology. Its hallmark finding is an erythema migrans (EM)–like skin lesion after a lone star tick (*Amblyomma americanum*) bite. Although it is considered self-limited, STARI is difficult to diagnose and manage because of characteristics similar to Lyme disease. STARI is not known to cause any sequelae after rash resolution; the role of antimicrobial drugs in proven cases is therefore undetermined (*1*).

Previous descriptions of STARI focused on the southern United States. Because lone star tick populations have increased in the mid-Atlantic and northeastern United States, regions highly endemic to Lyme disease, differentiating STARI from Lyme disease has become a challenge (*2*). We describe the case of a child evaluated in Long Island, New York, USA for an EM-like skin lesion. 

An 8-year-old girl with a history of asthma and allergic rhinitis living in eastern Long Island was brought in June 2025 for evaluation of a recent EM-like lesion. Approximately 2 weeks before her visit, she had a 5-cm annular lesion with peripheral erythema and a central nodule on the upper back (Figure 1). She also experienced low-grade fever, posterior cervical lymphadenopathy, and myalgia. A tick had been removed from the lesion site and stored in a sealed bag 12 days before the rash began. The patient did not receive doxycycline postexposure prophylaxis. Because early localized Lyme disease was suspected, her pediatrician prescribed a 2-week course of amoxicillin. The rash and associated symptoms resolved within 1 week. She was asymptomatic at the time of the follow-up consultation.

**Figure 1:**
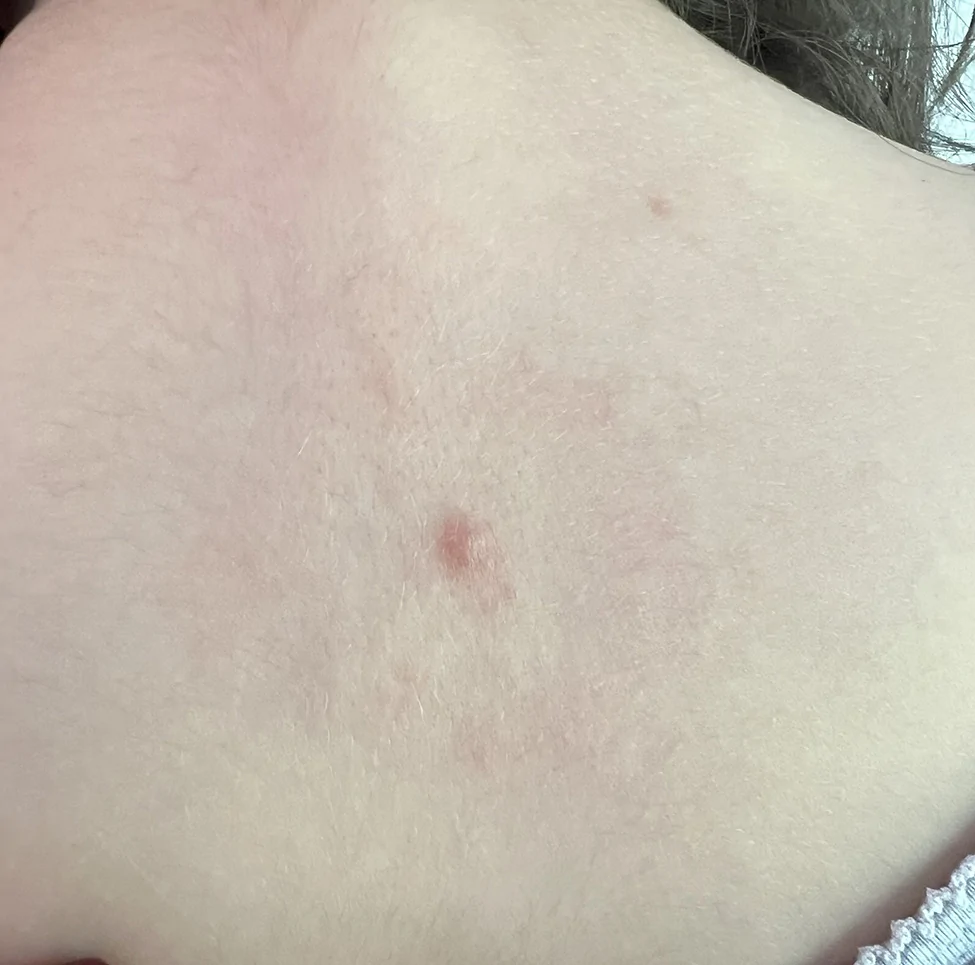
Erythema migrans–like skin lesion at the site of a tick bite on patient’s upper back in study of southern tick–associated rash illness in pediatric patient, New York, USA, 2025.

Although care providers initially suspected Lyme disease, the biting tick was identified as an adult male lone star tick (Figure 2), which does not transmit *Borrelia burgdorferi*, which mean the patient’s signs and symptoms were consistent with a diagnosis of STARI. We extracted DNA from the tick, performed nested PCR targeting the *Borrelia* flagellin gene (*3*), and confirmed the presence of a *B. lonestari*–specific sequence (GenBank accession no. PZ457021).

**Figure 2:**
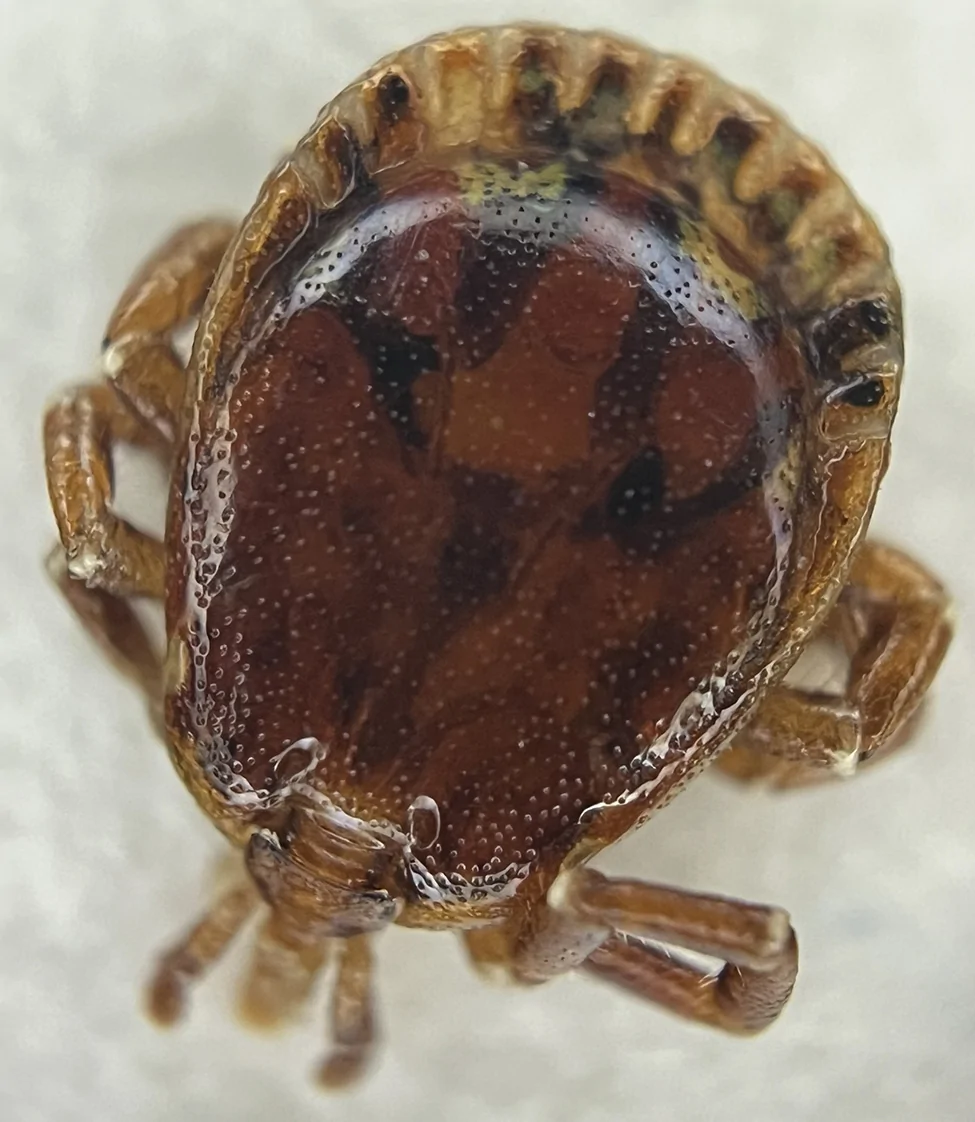
Adult male lone star tick (*Amblyomma americanum*) from study of southern tick–associated rash illness in pediatric patient, New York, USA, 2025. The tick was removed from the patient 12 days before rash onset.

*A. americanum*, an aggressive tick species, has expanded across the United States (*2**,**3*). In the US Northeast, diagnosing STARI is challenging because of a high rate of *B. burgdorferi*–infected *Ixodes scapularis* ticks (*2*). As *A. americanum* tick populations have increased across Long Island, the proportion of EM lesions caused by Lyme disease has decreased, likely because of higher STARI incidence (*2*). National STARI incidence remains unknown because the condition is not reportable and is often presumed to be the result of *B. burgdorferi* infection.

STARI was described in 1995 in 45 patients with EM-like lesions in Missouri, USA, in a region without Lyme disease (*4*). Study participants had negative serology results for Lyme disease and negative *B. burgdorferi* culture results from skin lesion biopsies, which raised suspicion of an alternative diagnosis (*4*). *B. lonestari* was identified as a potential cause of STARI after it was identified in both skin biopsy and the associated tick in a patient bitten by a female *A. americanum* tick in North Carolina (*5*). A subsequent Missouri study assessed *B. burgdorferi* and *B. lonestari* in skin biopsies from patients with EM-like lesions (*6*); results from 16S eubacterial rDNA PCR, *glpQ* PCR for *B. lonestari* DNA detection, and *B. burgdorferi* culture were negative (*6*). In a later study, 16s rRNA metagenomic sequencing of blood samples collected during 2018–2019 from STARI patients did not identify any tickborne bacteria (*7*). Consequently, it has been suggested that STARI is caused not by an infection but rather a hypersensitivity reaction (*8*), possibly related to alpha-gal syndrome, which is also caused by the bite of a lone star tick (*8*). *B. lonestari* prevalence on Long Island is uncommon (detected in 1% of *A. americanum* ticks) (*9*), which made identification in our case particularly interesting. Because our patient did not undergo testing for *B. lonestari* infection, we cannot determine if that bacterium caused her symptoms.

STARI diagnosis is difficult because it shares features with Lyme disease; however, subtle clinical differences exist. Compared with EM patients from New York, a highly Lyme disease–endemic state, patients from Missouri, who presumably had STARI, experienced a shorter duration from tick bite to skin lesion onset; had fewer associated symptoms; were less likely to experience multiple lesions; had lesions that were smaller, more circular, and had more central clearing; and recovered more quickly (*10*). Although withholding antimicrobial treatment might be reasonable in confirmed cases of STARI, doing so in *B. burgdorferi*–endemic regions poses the risk of undertreating Lyme disease. As our case demonstrated, STARI can be diagnosed only if a lone star tick is identified, underscoring the importance of preserving the biting tick.

The etiology of STARI remains unknown; whether *B. lonestari* is the causative agent is not clear (*5*,*6*). Although detection of *B. lonestari* does not establish it as the cause, our findings raise questions about *B. lonestari* pathogenicity and underscore the need for improved diagnostics for patients with EM-like lesions.
